# Multi-Infections of Feminizing *Wolbachia* Strains in Natural Populations of the Terrestrial Isopod *Armadillidium Vulgare*

**DOI:** 10.1371/journal.pone.0082633

**Published:** 2013-12-06

**Authors:** Victorien Valette, Paul-Yannick Bitome Essono, Winka Le Clec’h, Monique Johnson, Nicolas Bech, Frédéric Grandjean

**Affiliations:** 1 Université de Poitiers, UMR CNRS 7267 Ecologie et Biologie des Interactions, Equipe Ecologie Evolution Symbiose, Poitiers, France; 2 Laboratoire Biogéosciences – UMR CNRS 6282 - Equipe Ecologie Evolutive – Université de Bourgogne, Dijon, France; 3 Department of Genetics, Texas Biomedical Research Institute, San Antonio, Texas, United States of America; Centro de Pesquisas René Rachou, Brazil

## Abstract

Maternally inherited *Wolbachia* (α-Proteobacteria) are widespread parasitic reproductive manipulators. A growing number of studies have described the presence of different *Wolbachia* strains within a same host. To date, no naturally occurring multiple infections have been recorded in terrestrial isopods. This is true for *Armadillidium vulgare* which is known to harbor non simultaneously three *Wolbachia* strains. Traditionally, such *Wolbachia* are detected by PCR amplification of the *wsp* gene and strains are characterized by sequencing. The presence of nucleotide deletions or insertions within the *wsp* gene, among these three different strains, provides the opportunity to test a novel genotyping method. Herein, we designed a new primer pair able to amplify products whose lengths are specific to each *Wolbachia* strain so as to detect the presence of multi-infections in *A. vulgare*. Experimental injections of *Wolbachia* strains in *Wolbachia*-free females were used to validate the methodology. We re-investigated, using this novel method, the infection status of 40 females sampled in 2003 and previously described as mono-infected based on the classical sequencing method. Among these females, 29 were identified as bi-infected. It is the first time that naturally occuring multiple infections of *Wolbachia* are detected within an individual *A. vulgare* host. Additionally, we resampled 6 of these populations in 2010 to check the infection status of females.

## Introduction

*Wolbachia* are endosymbiotic α-Proteobacteria, closely related to the *Rickettsia*. *Wolbachia* are highly diversified and are currently divided into 11 supergroups (A to F and H to L, and supergroup G which is considered to be a recombination between A and B) [1-4]. They are mainly maternally inherited and infect a wide range of nematodes and arthropods [5-7]. Depending on both the bacterial lineage and the host, they may induce very diverse effects on host reproduction such as cytoplasmic incompatibility [8], male killing [9], thelytokous parthenogenesis [10], or feminization of genetic males [11]. All these manipulations enable the spread of *Wolbachia* by decreasing the expected productivity of uninfected females, or by distorting the sex-ratio in favour of infected females [12]. They can induce reproductive isolation, or even an alteration in host reproductive ecology [13-15]. As a result, many *Wolbachia* are considered to be parasites of reproduction and thus play a determining role in the infected hosts' evolution.

In 2008, Duron et al. [16] proposed that at least a third of arthropod species were infected by a diverse assemblage of maternally inherited bacteria and an important number of studies seems to indicate that, on both a population and individual scale, many of these cases of multiple infections involve different *Wolbachia* strains [17-19]. For instance, in the ant *Formica exsecta*, there can be up to five strains of *Wolbachia* within an individual host [12]. Thus, within a host, various interactions are expected to occur between coexisting symbionts and these will influence both the life history traits of the host and the dynamics of symbiont spread [20]. Theoretical predictions of either coexistence or exclusion of different strains suggest that if there are two *Wolbachia* strains inducing cytoplasmic incompatibility in a population with no co-infected individuals, the strain with the higher relative fitness will drive the other out of the population. However, in populations where co-infection in individual hosts is observed, uninfected, singly infected and co-infected hosts can co-occur. Within these populations, long-term persistence of co-infections may be possible, during which time both the parasites and the hosts are probably selected and evolve together to survive [21,22]. Moreover, Ironside et al. [23] proposed that the presence of two co-occuring feminising parasites in natural populations of *Gammarus duebenii* could be possible following either a recent invasion of a new parasite, a horizontal transmission of one or both parasites, or the spread of alleles for resistance to the most dominant parasite in host populations.

In terrestrial isopods (Crustacea, Oniscidea), *Wolbachia* induce cytoplasmic incompatibility in three species, *Porcellio dilatatus petiti* [24], *Porcellio dilatatus dilatatus* [25] and *Cylisticus convexus* [26] and feminization in many others, including members of the genus *Armadillidium*, such as *Armadillidium vulgare* [27] and *Armadillidium nasatum* [28]. In *A. vulgare*, two distinct feminizing *Wolbachia* strains (*w*VulC and *w*VulM) have been identified in various populations [29]. More recently, Verne et al. [30] showed that several natural populations of *A. vulgare* presented a third *Wolbachia* strain named *w*VulP. This latter strain showed evidence of recombination events between *w*VulC and *w*VulM that have occurred on the *wsp* gene [30]. Although multiple infections within a given individual host have never been observed *in situ*, the presence of different *Wolbachia* strains in the same terrestrial isopod host populations and the existence of recombination between feminizing strains suggest that co-infections are possible and expected. To date, few studies [31,32] have investigated the prevalence of *Wolbachia* in field populations of *A. vulgare*. Based on the classical sequencing method (amplification and sequencing of the *wsp* gene), these studies have failed to detect the presence of multiple infections. Indeed, in this case, only the main PCR product is generally detected. Thus, this classical methodology seems not suitable to detect multiple infections. Herein, we designed a novel method to detect and discriminate the three different *Wolbachia* strains known to infect *A. vulgare*. From the study of Verne et al. [30], we inferred that several insertion or deletion events have occurred within the *wsp* gene fragment. Thus, we designed a new primer set and, after amplification, different product sizes are expected with specific lengths for each *Wolbachia* strain. 

In this paper, we tested the methodology by performing experimental mono-, bi-, and tri-injections of different *Wolbachia* strains in *Wolbachia*-free *A. vulgare* hosts. Using this new method, we also re-investigated the work of Verne et al. [32] on the prevalence of *Wolbachia* strains in several natural populations sampled in 2003. Additionally, we resampled 6 of these populations in 2010 to follow the dynamics of *Wolbachia* strains' prevalence over time. With this new genotyping method, we reveal for the first time the occurrence of multiple infections of *Wolbachia* within individual *A. vulgare* hosts originating from natural woodlice populations. 

## Materials and Methods

### Ethic Statement

All experimental procedures and animal manipulations did not require an ethics statement.

### Authorizations for field sampling

No specific permissions were required for the 7 sampled locations which are public sites. No specific permissions were required for our activities. We confirm that the field studies did not involve endangered or protected species.

### A novel method to detect and genotype *Wolbachia* strains in *Armadillidium vulgare*

In order to discriminate the three *Wolbachia* strains known to infect *A. vulgare*, we designed a new primer pair able to amplify products whose lengths are specific to each *Wolbachia* strain. To this end, we aligned *wsp* sequences of each *Wolbachia* strain (about 600 bp) found in *A. vulgare* (*w*VulC, GenBank accession number: DQ778095; *w*VulM, GenBank accession number: DQ778097; *w*VulP, GenBank accession number: DQ778096). Primer 3® software [33] was used to design forward (5’TGGTGCAGCATATGTAAGCAA3’) and reverse (5’AAAACTTTGTGTGCGCCTTT3’) primers able to amplify a shorter PCR product (about 250 bp) which includes the variable region. PCRs were performed using a Trio-Thermoblock (BiometraGmBH) in a final volume of 12 μL [0.05 μL Taq polymerase (5 U/μL) (Promega), 2.5 μL of Taq buffer (5X), 0.5 μL of dNTP (8.3 mM), 0.5 μL of each primer (10 µM) and 1 μL of DNA template]. PCR cycling profile included an initial denaturing step of 5 min at 95°C, followed by 35 cycles of 30 s at 95°C, 30 s at 55°C, 1 min at 72°C, and a final step of 5 min at 72°C. The forward primer 5’TGGTGCAGCATATGTAAGCAA3’ was end-labelled with fluorescent phosphoramidite (6-FAM). The PCR products were run with the internal size standard GeneScan™- 500 ROX™ on an ABI PRISM 3130xl® automated sequencer. Allele sizes were scored using Genemapper® (Applied Biosystems). 

### Validation of the method by experimental mono- and multi-injections of *Wolbachia* strains in *A. vulgare*

In order to validate the methodology, *Wolbachia*-free female *A. vulgare* were injected with one, two or three strains of *Wolbachia*. The *Wolbachia* inoculates were obtained from host lineages, originating from 3 natural French populations that have been maintained in our laboratory for many years (the Méry-sur-Cher population harbours the *w*VulM *Wolbachia* strain; the St Cyr population harbours the *w*VulC strain; and the Poitiers population harbours the *w*VulP strain). One woodlouse lineage (from Nice, France) is *Wolbachia*-free and was used as a recipient for the experimental injections. Inoculates were obtained from the ovaries of 5 individuals from each woodlice line. The ovaries were crushed in 1 mL of Ringer buffer. The resulting suspensions were filtered through a 1.2 µm pore membrane to obtain inoculates. Using Quantitative-PCR, we estimated the *Wolbachia* concentrations for each inoculate to be 1.43 x 10^7^, 1.15 x 10^7^ and 3.24 x 10^7^ *wsp* copy numbers/µL for *w*VulC, *w*VulM and *w*VulP, respectively. *Wolbachia*-free females of *A. vulgare* (Nice line) were injected with 1 µL of inoculate containing either no *Wolbachia* (negative control), one of the three *Wolbachia* strains (*w*VulM, *w*VulC or *w*VulP), or an equal mix of either two different strains (*w*VulM/*w*VulC, *w*VulM/*w*VulP, *w*VulC/*w*VulP) or three *Wolbachia* strains (*w*VulM/*w*VulP/*w*VulC), using a 10 µL Hamilton needle adapted with a 1 mm glass capillary. Five females were injected for each treatment. They were placed at 20°C, at a light-to-dark photoperiod of 18:6, and dissected 28 days later in order to isolate their ovaries from which we extracted DNA using the protocol described in Kocher et al. [34]. Moreover, DNA from each inoculate was also extracted. We amplified all of these DNA samples with the newly designed primer pair in order to compare and verify, on an ABI PRISM 3130xl® automated sequencer, the sizes of the amplified fragments. This PCR reaction was carried out in the same conditions as above and was qualified as the 'novel genotyping method' for *Wolbachia* strain detection in *A. vulgare*.

### Field study

In 2003, Verne et al. [32] sampled 7 populations in the West of France. In these populations, the classical sequencing method revealed that, among 124 analyzed females, 40 were mono infected by *Wolbachia* (i.e. 7 females were infected by *w*VulM, 5 females by *w*VulP and 28 by *w*VulC) (Table 1). We used our novel genotyping method in order to re-investigate the infection status of these females [32]. Moreover, in order to estimate the evolution dynamics of the different *Wolbachia* strains in natural populations of *A. vulgare*, we resampled, in 2010, 6 of the 7 populations previously analyzed in Verne et al. [32]. We collected 85 females, extracted the DNA from ovaries following the protocol described above and then characterized the infection status using the novel genotyping method (Table 1). 

**Table 1:** Prevalence of *Wolbachia* strain infection in natural populations of *Armadillidium vulgare* sampled in 2003 and 2010.

**Location**	**Sampling year**	**Sex ratio (♂/♀)**	**Number of analyzed females**	**Number of infected females (*%*)**	**Number of females infected by different Wolbachia strains**				
					***w*VulM**	***wVulC***	***wVulP***	***wVulM / C***	***wVulP / C***
**Ensoulesse** 46°38'6.00262’’N 00°23'30.63477’’E	2003	1.11	9	2 (22.2)	0	0	2 (22)	0	0
	2010	1.13	20	11 (55)	0	0	11 (55)	0	0
**Poitiers** 46°35'3.77006’’N 00°22'16.07919’’E	2003	1.38	8	8 (100)	1 (12.5)	1 (12.5)	4 (50)	2 (25)	0
	2010	0.40	17	17 (100)	0	1 (6)	11 (65)	4 (23)	1 (6)
**Coulombiers** 46°29'18.92092’’N 00°11'31.56164’’E	2003	0.36	22	3 (14)	0	0	0	3 (14)	0
	2010	0.90	12	5 (42)	0	2 (17)	0	1 (8)	2 (17)
**Saint Maixent l'Ecole** 46°24'58.01243’’N 00°11'56.61584’’W	2003	0.17	35	18 (51)	0	0	0	18 (51)	0
	2010	0.79	12	4 (33)	0	3 (25)	0	1 (8)	0
**La Crèche** 46°21'40.08011’’N 00°18'21.95247’’W	2003	0.77	22	3 (14)	0	0	0	3 (14)	0
	2010	0.93	12	7 (58)	0	4 (33)	0	2 (17)	1 (8)
**Beauvoir-Sur-Niort** 46°10'35.91493’’N 00°28'30.45661’’W	2003	0.89	18	3 (17)	2 (11)	0	0	1 (6)	0
	2010	0.47	12	7 (58)	0	2 (17)	1 (8)	4 (33)	0
**Granzay-Gript** 46°12'52.08761’’N 00°28'6.57765’’W	2003	0.42	10	3 (30)	1 (10)	0	0	2 (20)	0
	-	NA	NA	NA	NA	NA	NA	NA	NA

Results are obtained using the novel genotyping method. Sampled locations, their GPS coordinates (longitude and latitude in the World Geodetic System 1984 (WGS 84)), sampling year, sex ratio (♂/♀), number of analyzed females , number of infected females (percentage) are indicated in the table. Granzay-Gript was not sampled in 2010 (NA=not available).

## Results

### Validation of the methodology

**Figure 1:**
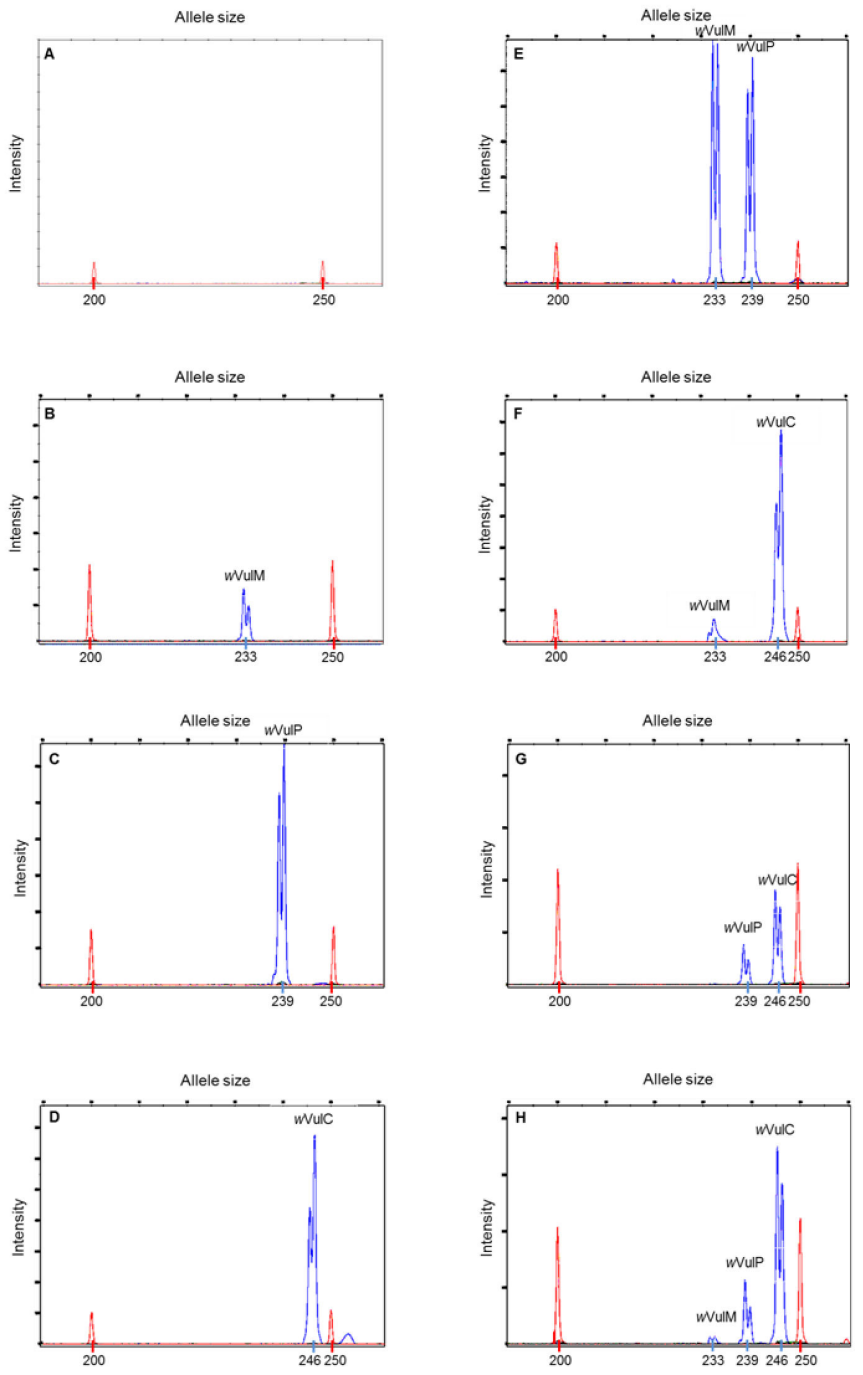
Chromatograms obtained from experimental injections of the different *Wolbachia* strains. Chromatograms are obtained respectively when inoculate was made up of: A) no *Wolbachia* strain; B) *w*VulM strain; C) *w*VulP strain; D) *w*VulC strain; E) *w*VulM and *w*VulP strains; F) *w*VulM and *w*VulC strains; G) *w*VulP and *w*VulC strains; H) *w*VulM, *w*VulP and *w*VulC strains. Size markers appear in red. *Wolbachia* appear in blue. The fragment sizes for *w*VulM, *w*VulP and *w*VulC are 233, 239 and 246 bp respectively.

Both primers designed from the alignment of *Wolbachia* strain wsp sequences gave specific amplified fragments for each strain. Thus, we obtained amplification products of 233, 239 and 246 base pairs for *w*VulM, *w*VulP and *w*VulC, respectively. PCR amplification of the inoculate obtained from the *Wolbachia*-free females (Nice line) gave no amplification product. Results from the injection experiments showed patterns in accordance with the number and the size of injected strains. Whatever strain, one peak was observed on Genemapper® when inoculate was made up of only one strain. Two peaks were observed for doubly injected individuals, and three peaks were observed for the individuals injected by inoculate containing the three strains (Figure 1). No peak was observed when the individuals were injected by *Wolbachia*-free inoculate. 

### *Wolbachia* prevalence and dynamics of infection in natural populations

The results obtained using the novel genotyping method showed a very high prevalence of bi-infected individuals. Indeed, in 2003, classical sequencing analysis revealed 40 mono-infected females [32] whereas, from the same females, the novel genotyping method identified only 11 females as being mono-infected (i.e. 27.5%) and 29 females as being infected by both the *w*VulC and *w*VulM strains (i.e. 72.5%). Among the 11 mono-infected females, 4 harboured *w*VulM (observed in Poitiers, Beauvoir-sur-Niort and Granzay-Gript), 1 *w*VulC (observed in Poitiers) and 6 *w*VulP (observed in Ensoulesse and Poitiers).

In the comparative sampling carried out in 2010, 51 females on the 85 analyzed were infected by *Wolbachia* (Table 1). Among these, our method reveals that 35 individuals were mono-infected (12 *w*VulC and 23 *w*VulP) and 16 individuals were bi-infected (12 *w*VulC/*w*VulM and 4 *w*VulP/*w*VulC). No females harboured the *w*VulM strain alone (Table 1). We detected no bi-infections involving *w*VulP and *w*VulM, regardless of both the population and the sampling year.

## Discussion

### Multi-infections in *A. vulgare*

The experimental injections of different *Wolbachia* strains performed in the present study revealed that genotyping allows an evident discrimination of the three strains in *A. vulgare* which are characterized by specific amplified fragment sizes. This methodology is also very efficient to reveal multi-infections in *A. vulgare* from both experimental strain injections and individuals sampled in the field. Verne et al. [32] reported only mono-infected females based on sequencing analyses. Here, using the novel genotyping method to reanalyze the same samples, it would appear that multiple infections in *A. vulgare* are rather common with high proportions of bi-infected females (72.5%). Although co-infection of different *Wolbachia* strains in a single individual is commonly found in arthropods [18,35-38], this is the first time that doubly infected individuals have been observed in natural population of terrestrial isopods. This result is not really surprising as several recent studies have suggested that horizontal transfers of *Wolbachia* in *A. vulgare* may explain both the discordance between *A. vulgare* and *Wolbachia* phylogenies [32] and the presence of the recombinant strain *w*VulP [30]. Indeed, for recombination to occur, two strains need to be in close contact. Such proximity is possible if an individual host is infected by several strains. Previous studies have reported that haemolymph contact, predation and parasitism are possible routes for horizontal transfers of *Wolbachia* in *A. vulgare* [39-41]. Haemolymph contacts may be more frequent than previously thought, due to the fact that woodlice populations are often densely populated, and because of the abundance of injured individuals as a result of predations [42,43] or incidents during molting [39]. Thus, a given *Wolbachia* strain could spread through a population through such horizontal transfers and infect individuals already infected by another *Wolbachia* strain. 

In 2003, all of the bi-infected females contained both the *w*VulC and *w*VulM strains but no bi-infections involving the *w*VulP strain was observed. This result is consistent with the prevalence of these strains in natural populations. Indeed, the *Wolbachia* strains *w*VulC and *w*VulM were more frequently observed *in situ* than the *w*VulP strain [32,40]. 

### The Co-infection: a transition phase?

In theory, the co-existence of several feminizers within the same individual is unstable at equilibrium [44]. When two feminizing *Wolbachia* strains are in competition within the same host, the strain with the higher fitness is fixed [45]. Based on our results, it is difficult to give any firm conclusions concerning the evolution of *Wolbachia* strain prevalence between 2003 and 2010 , but we can expect that the *w*VulC strain will progressively replace the *w*VulM in the near future. Indeed, according to Cordaux et al. [29], *w*VulM is considered as a resident strain, with a transmission rate to the offspring lower than that of *w*VulC, this last strain being considered as an invasive strain. Recent experimental studies from challenged woodlice reveal that *w*VulC has a higher development rate than *w*VulM within the host tissue, suggesting that *w*VulC strain could be the most virulent and dominant strain (Johnson, unpublished data). 

The *w*VulP *Wolbachia* strain is considered to be a recent strain resulting from the recombination of *w*VulC and *w*VulM [30]. According to evolutionary theory, it is expected that this strain would have a higher fitness than the others, leading to an increase in its prevalence in natural populations.. A follow up of the *A. vulgare* populations and their *Wolbachia* infection status could allow us to verify this hypothesis. 

## Conclusion

One of the main problems in the detection and characterization of different *Wolbachia* strains in *A. vulgare* was the absence of a rapid, inexpensive screening tool. Herein, we describe a novel PCR-based approach allowing the discrimination between *w*VulC, *w*VulM and *w*VulP on the basis of different amplification sizes by genotyping. For the first time, our study reports the presence of multiple *Wolbachia* strain infections in natural populations of *A. vulgare*, suggesting that such multiple infections are much more frequent than previously thought. Whether the presence of two different *Wolbachia* strains in a single individual is the result of horizontal transfer, hybrid introgression or co-divergence, as has recently been shown in other species complexes, awaits investigation although elements here support the idea of horizontal transfers. Additionally, three species closely related to *A. vulgare*, *A. tunisiense*, *A. pelagicium, A. nasatum* have showed amplification pattern corresponding to mono infected individuals suggesting that this technique could be efficient to check the infected status in several isopods species.

The method presented here offers new perspectives in the detection of multiple infections in natural populations of *A. vulgare* and related species and will also be invaluable in studies of infection dynamics after micro-injections of several strains in *Wolbachia*-free female hosts. 
